# Report from the Sanitary Committee

**Published:** 1855-07

**Authors:** 


ART. VI.—*Report from the Sanitary Committee*, Appointed at the Annual Meeting of Cook Co. Medical Society, held on the first Tuesday of April, 1855—General Observations—Cleanliness of the City—General Condition of the Good Health—Cholera, its connection with temperature and humidity—Tubercular Consumption, &c., &c.

THE resolution requiring the appointment of a Committee to report monthly on the Sanitary condition of Chicago and the progress of disease therein, was first adopted at the Annual Meeting of this Society in April, 1854. And though its reports were not made with perfect regularity, yet they presented a connected history of diseases in the city during the year, and seem to have been of sufficient interest to cause a renewal of the Committee for another year. There having been no meeting of the Society on the first Tuesday in May, the present report will include two months, from the first of April to the first of June inclusive.

Having been absent from the city part of this time, I must rely more than usual upon the observations of others, and can only speak in general terms of the meteorological phenomena of the last two months. With the exception of two or three days during the third week in April, the temperature has been unusually low, so much so as to retard much the progress of vegetation, and cause the spring to be denominated an unusally backward one. There has been less than the average fall of water during the two months and the atmosphere has been more dry than usual. Almost all our rains, too, have been followed by a clear, cold, and dry atmosphere. In all these respects the past two months present a marked variation from the same period in 1854. (See report to this Society, published in the N. W. Med. and Surg. Journal, June, 1854.)

In regard to drainage, cleanliness of streets, and general sanitary regulations, the city remains in substantially the same condition as during the two past years. I cannot learn that the period under consideration has been marked by the prevalence of any particular form of disease, but on the contrary, all concur in representing the general health of the city as having been very good. The city sexton reported the whole number of deaths in April to be 105, and in May 90.

This, for the latter month especially, presents a ratio of mortality more than 50 per cent. less than for the corresponding month last year, and corresponds closely with that for May, 1853, when the city enjoyed a remarkable degree of good health during the entire year. I have been able to learn of but one well authenticated case of cholera, during the past two months, and that occurred in the person of a sailor who had come up the Mississippi River direct from New Orleans, and who was attacked before he reached the city. The symptoms were characteristic and strongly marked, and the case proved speedily fatal. The patient on his arrival was received into a boarding house on Kinzie st., where he was in contact with several other persons. No other cases occurred at the place either then or since.

For the facts in this case I am indebted to Dr. De Laskie Miller, under whose observation it occurred. It occurred on April 28, about the same time that the disease broke out on boats crowded with emigrants on the Upper Mississippi River; and I place it on record as an item that may be of importance hereafter. The philosophical physician, who is ever searching for the causes of disease, whether exciting or predisposing, will not fail to compare the months of May, 1853–4–5, with much care and interest.

In every visible particular the population and general sanitary condition of our city presented the same aspects during the month in each of the years named. And yet during the first and the last the population of our city enjoyed unusually good health, while during the intermediate one was developed the beginning of one of the most severe epidemics to which our city has ever been subjected. Those members of the Society who have attentively noted the meteorological character of the several seasons named will not be at a loss for an adequate cause to explain the difference. The observations made on an extensive scale by the Smithsonian Institute, as well as those gathered from private sources, showed that the spring of 1853 was characterized by an atmosphere containing less than the average amount of moisture, and was throughout the country accompanied by a low ratio of mortality. From personal observations concerning the condition of the atmosphere during the spring and summer of 1854, I was enabled to report, from time to time, to this society, the fact that the atmosphere from early in April to the latter part of summer, contained more than the ordinary degree of moisture, often indeed amounting almost to saturation, and that there was a very close relationship between the degree of atmospheric heat and moisture combined, and the prevalence of cholera. At the recent annual meeting of the American Medical Association, a very interesting and elaborate report was made by Dr. S. B. Hunt, of Buffalo, on the agency of meteorological changes in the production of disease, in which he pointed out the same relationship as being distinctly visible during the prevalence of cholera in that city in the summer of 1854. I have called attention to this subject now, not for the purpose of entering upon any detailed investigation, but to excite if possible a larger number of members to make daily memorandums both of atmospheric conditions and the prevalence of disease. It is as necessary to do this during seasons of good health as during those characterized by the unusual prevalence of disease. Otherwise the elements of comparison cannot be obtained with that fulness necessary to ensure accuracy of deduction. Again, I have stated, as an exception to the general low temperature of the two past months, that two or three very warm days occurred during the third week in April. The warmest of these days were the 17th and 18th of April, when in this city the mercury rose to 86 degrees F. As near as I have been able to procure dates, it was during this and the following week that the cholera appeared suddenly and fatally on several boats on the upper Mississippi. Being absent from the city at the time I made no note of the atmospheric moisture during that period. If any member of the Society possesses knowledge on that point, he would confer a great favor by reporting it.

Of the 195 deaths which have occurred in the city during the last two months, 35, or more than one sixth of the whole, are reported to have occurred from *Consumption*. We thus see, here, as almost everywhere else in christendom, the ravages of a disease which, though accompanied by none of the terrors incident to the raging pestilence, yet annually carries more victims to the grave than cholera and yellow fever combined. And I allude to it now solely for the purpose of earnestly soliciting each member to note down the following inquiries, and carefully procure answers to them in reference to each case that may come under observation during the coming six months at least:

1st. Are there traces of hereditary tubercular influence?

2d. At what age did the patient show symptoms of tubercularization?

3d. Was the patient accustomed to eat *fat meat* of any kind after the age of ten years, and if so, to what extent?

4th. Was the patient accustomed to use alcoholic drinks, either fermented or distilled, at any period of life and to any extent, and if so, of what kind?

5th. Since the full development of the disease, has the patient used either alcholic drinks or Cod-Liver Oil as remedies, and if so, with what effect?

Carefully elicited answers to these queries, obtained from a large number of cases and by different members of the profession, might lead to valuable deductions.

Some of them have been suggested by the doctrines inculcated in a paper read before the last annual meeting of the American Medical Association, by Prof. Charles Hooker, of New Haven, Con.

The general idea inculcated by Dr. Hooker, seemed to be that the tubercular diathesis consisted in a defective assimilation of carbonaceous matter, especially that in the form of oils or fat meat. And he made the general statement, that persons who were in anywise disposed to scrofulous or tuberculous disease, and who did not acquire a good relish for the daily use of fat meat before the age of fifteen years, generally died from consumption before 35 years. It is also well known, at the present time, many appear to regard the use of alcoholic liquors as more or less efficacious in counteracting the tendency to tubercular disease. So far as I can learn, all these ideas in regard to alcohol, the oils, and other carbonaceous matters, are founded on no definite or positive data, and consequently are nothing more than vague individual opinions or hypotheses.

